# Report of the Buffalo General Hospital

**Published:** 1864-08

**Authors:** 


### ART. IV.—*Report of the Buffalo General Hospital.*

The following is, with some changes and additions, taken from a Report prepared by a committee of the Medical and Surgical Staff of the Buffalo General Hospital, of which Drs. P. H. Strong, (Chairman,) J. Hauenstein, and J. R. Lothrop were members. The statistics of the Report, from which extracts are taken, were prepared by Dr. John N. Brown, present House Physician and Surgeon. The deficiencies are not chargeable upon him, they existed in records left by his predecessor.

The number of cases received at the Buffalo General Hospital for treat. ment during the year ending July 1st, 1864, was 442. Of these 57 were under treatment, at the time the report was made, and as results cannot as yet be arrived at, the above number is not included in the following statement.

After the deduction, there remain 385 cases of which the results can be stated as follows in a general way:

| | | |
|---|---|---|
| Cases under treatment | | 385 |
| Discharged cured | 193 | |
| Discharged more or less benefited | 119 | |
| Discharged not benefited | 23 | |
| Died | 50 | —385 |

Classifying the diseases as simply as possible, with some degree of distinction, the statement may be thus made:

*Diseases of the Nervous System.*

| | |
|---|---|
| Apoplexy | 1 |
| Softening of the Brain | 1 |
| Paralysis | 3 |
| Sciatica | 2 |
| Delirium Tremens | 17 |
| Insanity | 2 |
| Epilepsy | 4 |
| Hysteria | 1 |

*Diseases of the Abdomen and Abdominal Organs.*

| | |
|---|---|
| Peritonitis | 4 |
| Diarrhœa | 20 |
| " Chronic | 25 |
| Dysentery | 6 |
| Enteritis | 2 |
| Jaundice | 3 |
| Hernia, inguinal | 2 |
| Fistula in Ano | 3 |

*Diseases of the Skin and Cellular Tissue.*

| | |
|---|---|
| Abscess | 2 |
| Furunculus | 4 |
| Ulcers | 6 |
| Erysipelas | 2 |
| Eczema | 2 |
| Scabies | 1 |
| Varicose Veins | 3 |

*Fevers.*

| | |
|---|---|
| Intermittent | 16 |
| Remittent | 2 |
| Typhoid | 18 |

*Diseases of the Respiratory Organs*

| | |
|---|---|
| Bronchitis | 10 |
| Pneumonia | 16 |
| Pleurisy | 4 |
| Phthisis | 13 |

*Diseases of the Genito-Urinary Organs.*

| | |
|---|---|
| Vesical Calculus | 1 |
| Bright's Disease | 1 |
| Gonorrhœa | 5 |
| Syphilis, primary | 22 |
| " secondary | 11 |
| Dysmenorrhœa | 2 |
| Ulceration of Os Uteri | 2 |

*Diseases of the Bones and Joints.*

| | |
|---|---|
| Inflammation of Knee Joint | 1 |
| Disease of Hip Joint | 1 |
| Dislocations, ankle 1, shoulder 2 | 3 |
| Caries and Necrosis | 5 |
| Fractures | 9 |

*Diseases of the Eyes.*

| | |
|---|---|
| Conjunctivitis | 8 |
| Ophthalmitis, (traumatic) | 2 |
| Iritis | 3 |
| Cataract, (double) | 2 |

*Other Diseases.*

| | |
|---|---|
| Diphtheria | 4 |
| Opium Poisoning | 1 |
| Mumps | 1 |
| Frost Bite | 1 |
| Gangrene, (traumatic) | 1 |
| Debility | 10 |
| Cancer | 5 |
| Amputations | 2 |
| Rheumatism, acute | 10 |
| " chronic | 13 |
| General Dropsy | 1 |
| Parturition | 5 |
| Gunshot Wounds | 42 |
| Injuries | 7 |
| Tumors | 7 |

It would be desirable to state more in detail in many cases. This will be done as far as data are given, but in many cases there are no records from which to make more definite statements. Thus in the cases of pneumonia no statement can be made as to which lung was affected, when but one was invaded, or in how many cases both were involved. Neither can it be stated which side was involved in the cases of pleurisy, or which lung or part of lung was most seriously invaded in the cases of tuberculosis. Such deficiencies are owing to want of care in the record.

*Paralysis.*—Three cases of paralysis are mentioned. Of these, all three were cases of paraplegia. One, supposed to have been caused by inflammatory softening of the spinal chord, one from affection of the bones of the spine, originating from constitutional causes, and likely to terminate in recovery with deformity, and one caused by gunshot wound, ending in death.

*Diarrhœa.*—The cases of diarrhœa, of which quite a large number was under treatment, occurred mostly in soldiers, returning from the lower Mississippi, who had participated in the campaign in that region under Gen. Banks, which terminated with the surrender of Port Hudson. As the regiments progressed on their way to New England, those too sick to go farther were left. Many were therefore brought to the hospital. Their cases excited great interest, both from their nature and origin. They were generally slow to yield to treatment, and many proved fatal. Some of them were recent cases, the disease having made its first appearance on the passage up the river. They occurred in men worn down by hard service and poor food, and unless some improvement was manifest in a few days, were uniformly fatal. The lesion discovered by autopsies was generally a thickened, softened and contracted condition of the colon, particularly of the descending colon. It had a grayish appearance and pulpy consistence. In some cases the lower end of the small intestine was contracted and in a state of congestion or inflammation. When the disease had continued any length of time remedies were found to have but little influence upon it. The discharges in most cases were copious, frequent, yellowish and offensive, though there was some variation, they being in some cases bloody, and often attended with great pain. More than half the cases proved fatal.

*Hernia.*—One was a case in which an operation to relieve strangulation had been performed before the patient was sent to the hospital. At that time the incision, and part of the scrotum were in a sloughy condition, a portion of the intestine had given way, and fecal matter escaped through the incision. In this case recovery seemed probable, as the wound began to close, and fecal matters passed by the rectum. The opening into the ntestiue must have been small, and the caliber not much affected by cicatrization.

*Syphilis.*—The majority of the cases were chancroids, in which only local treatment was resorted to. Some of the cases were mixed, as for instance one in which a sore resembling a chancroid existed on the penis, and at the same time the beginning of a secondary eruption, which must have arisen from a previous infection, though the patient denied any knowledge of it. The skin affection soon developed into a very extensive and marked form of rupia, with very large, prominent crusts on the face and head, and the upper and lower extremities. Rupia usually is a late manifestation of syphilitic infection, and also indicates a thorough contamination, together with much impaired general health, calling for generous diet and tonics as well as mercury.

*Varicose Veins.*—These cases are mentioned in this connection for the reason that no other diseases of the blood vessels are recorded. In two of these cases obliteration of the vein was successfully practiced by means of injection of a solution of the persulphate of iron.

*Necrosis and Caries.*—Three cases of necrosis and two of caries. In one case the ramus and angle of the jaw with the articulating head was removed. It occurred in a scrofulous boy. It resulted in deformity, owing to interference with the growth of the jaw. In two other cases the necrosis was the result of injury, one a gun-shot wound, apparently perforating the femur near its middle without fracture, perhaps splitting it. In both the bone was in process of separation. One case of caries involved the knee joint, and led to amputation; the other was a case of Pott's disease, terminating fatally.

*Fractures.*—The cases of fracture were two of fracture of the skull. In one, fracture of the os frontis with detachment and depression of a piece of bone an inch in diameter. Trephining was practiced and the bone removed. This was followed by hernia cerebri and death.

Fracture of rib—simple.

" of neck of the femur.

" of tibia and fibula, compound, and resulting in amputation after a trial to save the leg.

Fracture of second phalanx of great toe, street railway accident, resulting in amputation.

Fracture of the femur in middle third, oblique, caused by fall from a house; result union, with slight shortening.

Fracture of tibia at lower third, transverse; result non-union at first. Union caused by perforating the ends of bone after the method of Dr. Brainard.

Fracture of fibula with partial dislocation, by turning the foot outwards. Site three inches above joint. Patient died of delirium tremens.

*Conjunctivitis.*—These include cases in which the worst results some times take place—impairment of vision from opacity of cornea, or even affection of the inner structures, adhesion of the iris, etc. In one case an operation was made for artificial pupil upon each eye at separate times, with the results of improving vision somewhat in both eyes. The operation consisted in each instance of the removal of a piece of the iris drawn out through a small opening in the cornea.

*Ophthalmitis.*—In each case one eye only was affected, by the penetration of a foreign body. Result, formation of pus in the eye-ball, rupture of the cornea and discharge of the lens and a portion of the liquid contents, with no sympathetic affection of the other eye.

*Cataract.*—These were cases of double cataract in persons fifty years of age. Operation by breaking up, the needle being introduced through the sclerotica. In one case tolerable vision was obtained. In the other, one was in a fluid condition, and the first puncture of the needle filled the anterior chamber with a milky fluid. Severe neuralgic pain and constant vomiting followed, only allayed by evacuation of the contents of the anterior chamber, after about twenty-four hours. Opacity of the cornea resulted. The other lens was being slowly absorbed with prospect of fair vision.

*Rheumatism.*—No case of cardiac complications is recorded. It seems hardly possible that so many cases of this disease, some of which were fatal, could have been unattended by a complication so frequently met with. The record must appear to be deficient in this particular.

*Diphtheria.*—Several cases of this disease occurred in the soldiers above mentioned. It came on in some whose prospect of recovery appeared good, and of course rapidly brought about a fatal termination. All but one died.

*Gun-Shot Wounds.*—The greater number of these were received in battle, the majority being bullet wounds. A few cases of wounds by fragments of shell were among them. One or two were accidental, occurring in civil life. The majority were flesh wounds, though some involved the bony structures. Two cases are recorded without mention of the part wounded. The parts wounded were,

| | | | |
|---|---|---|---|
| Abdomen | 1 | Back | 1 |
| Shoulder | 3 | Finger | 2 |
| Leg | 6 | Thigh | 6 |
| Arm | 6 | Face | 3 |
| Hip | 3 | Chest | 1 |
| Hand | 3 | Elbow | 1 |
| Foot | 7 | | |

The course of the ball and the escape of important vessels, were often quite remarkable. In one case the ball entered near the upper and inner side of the humerus, just escaping the brachial, and made its exit through the centre of the scapula. Several examples were observed of balls entering the wrist and passing up the arm, escaping near the elbow. Several wounds were caused by balls passing directly through the foot, the soldier being at the time flat on his back. A case of gun-shot wound of the tarsus was by the passage of a ball through the bones near the joint. Gangrene of the foot resulted, from loss of circulation and probably of transmission of nervous influence; the vessels and nerves of the foot being involved in the wound. An attempt to save life by amputation of a part of the tarsus was unsuccessful, death following. In one case the index finger was perforated near the extremity, the nail and part of the distal phalanx being carried away, leaving a round opening through the end of the finger. In the wounds of the thigh, in three or four cases, the ball was arrested by striking the bone, not fracturing it. In one a minie ball was completely flattened on the femur, producing no apparent injury, and was removed at the hospital. In another the ball probably passed through the femur, splitting but not producing complete fracture. There were few cases in which the ball was deflected from its course.

In one wound of the face a portion of superior maxillary containing the incisors and the bicuspids, one or two molars, and part of the floor of the nasal cavity was carried away. In another the ball entered about opposite the second molar in the upper jaw, and made its exit under the opposite ear, just escaping the large vessels of the neck. In one case the ball entered the elbow joint, separating the olecranon, and a portion of the articulating surface of the ulna, and exposing the rounded articulating surface of the outer condyle of the humerus. The loose bones and the ball being removed the wound closed with partial anchylosis.

*Injuries.*—Under this head are recorded several cases of injury from various causes, except by gun-shot wounds.

| | | | | | |
|---|---|---|---|---|---|
| There were of the wrist | 2 | | There were of the foot | 1 |
| " " " hand | 1 | | " " " breast | 1 |
| " " " knee | 1 | | " " " scalp | 1 |
| " " " ankle | 1 | | | |

The injuries to the wrist were light, viz; one by a circular saw on the inner side, lacerating the integument considerably, but leaving the tendons unhurt. One from a blow causing inflammation and swelling of the bursa on the inner side, so that fluctuation was distinct both above and below the annular ligament, extending beneath it. After some time it yielded to poultices, iodine and blisters.

The injury to the knee was more serious. It was a railroad accident. The joint was opened and the lower end of the femur split. After an attempt to save the limb, amputation of the femur was finally resorted to and followed by recovery.

The injury to the ankle was a street railway accident, the car wheel passing over the ankle, crushing both malleoli; the patient left the hospital soon after admission; the result, however, was ultimately the saving of the foot.

The injury to the foot was also a street railway accident, the wheel passing over the foot lengthwise on its inner side. The heel of the boot saved the heel and tarsus, but the metatarsal bones were crushed and the great toe lost by mortification. Abscess followed under the plantar fascia. Result, recovery, with some distortion of foot.

The injury to the breast was inflicted by a blow, and followed by abscess. Recovery took place after the pus was evacuated by incision.

The injury to the scalp was received in an accident by machinery; the clothes were caught in a belt and the boy made several revolutions, the head striking and suffering severe laceration of the scalp, but no fracture of bone. The injury was, however, so severe, that a large exfoliation of the outer table followed on the parietal prominence. Cicatrization at length took place.

*Tumors.*—Of the seven tumors, six were removed by operation. One was a uterine tumor, not interfered with. Five of the tumors removed were upon the neck, and one in the breast. The tumors of the neck were one lipomatous, situated on the back of the neck; three encysted being made up of several cysts or lobules, the contents being semisolid, situated on the side of the neck over the great vessels; one a single cyst with fluid (meliceratous) contents situated over the trachea and having but slight connection with it.

Of the tumors of the neck, four were successfully removed. The fifth was followed by death in twenty-four hours. It penetrated deeply into the neck, passing downwards behind the clavicle.

*Cancers.*—Two of the lip, both removed; in one case, with perfect closure of the wound; in the other, imperfect cicatrization and return of disease in a short time. Two of the uterus, and one of the breast; removal and cicatrization in the last so far successful; time only can settle the question of re-appearance.

*Amputation.*—Those mentioned refer to one of the leg and one of the arm, which were made prior to admission and required only ordinary care. The surgical operations performed during the year will be mentioned hereafter.

*Hospital Gangrene.*—Two distinct attacks of hospital gangrene occurred during the year; one in July, 1863, and the other began in June, 1864, and was progressing at the time of this report, which makes up the year to the end of June. The first attack came on at a time when there were no gun-shot wounds or wounded soldiers in the hospital. The second began in the wounds of soldiers, but affected also other wounds; one boy who had lost a finger by a pistol accident was attacked by it as well as the soldiers wounded by gun-shot wounds. It was not in either instance brought into the hospital. All wounds were healthy, or at least not in a state of gangrene, when the patients were received. In the first epidemic, three severe cases occurred. One boy had suffered an amputation of the thigh, and cicatrization was nearly completed. The patient was about the wards dressed. Suddenly the stump became painful, red and tender. With these appearances was high febrile excitement. Soon a small spot became elevated and grayish, and in a short time the whole cicatrix gave way and the end of the stump was open with a gray, sloughy appearance and raised edges. Eventually the stump became healthy and the skin closed over the bone, making a less perfect stump than at first. The second case occurred in an old man who had suffered a great loss of integument on the leg and thigh by sloughing after an accident—the passing of a wheel over the limb lengthwise, without causing fracture. The destruction of integument was so great that complete cicatrization was not looked for, but healing had so far progressed that nearly two-thirds of the original loss had been repaired and the remaining surface was healthy. On the 4th of July he walked, using the limb more, probably than was prudent. Soon after a raised grayish spot appeared just below the knee and enlarging quite rapidly, the whole surface originally involved became sloughy and the edges raised. This case soon terminated fatally, great febrile excitement or constitutional irritation attending the outset and progress of the disease. The third case affected the wound attending a compound fracture of both bones of the leg. A good deal of suppuration was going on about the ends of the bones, but there were healthy granulations about the wound and the general condition of the patient was good. While the other cases were progressing, the wound in this case became sloughy, hæmorrhage took place, the foot lost its nutrition and amputation was resorted to, resulting in healthy cicatrization of the incisions. These cases had a local origin and were in no way caused by the nature of the wound, or peculiar condition of the persons in whom the disease occurred; i. e. they were not gun-shot wounds, nor were the patients soldiers, whose general condition was that of depression from fatigue, suffering and unhealthy camp life. The cause was atmospheric probably, though one case may give rise to others. The second epidemic, though mostly confined to soldiers may have and probably did arise from the same causes. It was however observed that when gangrene attacked recent wounds the progress was less rapid and the destruction less extensive, than when the wound had mostly healed. The progress and destruction were in direct ratio to the amount of new tissue which had been formed in the process of cicatrization. Sloughing seldom was arrested till all new tissue had given way.

As far as treatment was concerned, the first cases were treated by the solution of chlorinated soda. The cases in the second epidemic had been only subjected to treatment by turpentine, which in several cases appeared to act well. As many cases have since arisen and various methods of treatment have been adopted, the report of the present year will contain a fuller statement of results and the comparative value of the different remedies used locally, for as to general management there can be but one course, viz, to support the patient by nutritious food and stimulants, and of course the very commonest and least intellectual routine would hit upon quinine as an important medicinal agent. The manner in which the suggestion for the adoption and the inquiries as to the employment of this very important and we may say very well known drug, are made, often impress one with the idea, that the properties and uses of the substance are facts known only to, and in fact discovered by the individual recommending it. The probability is that the disease can be spread by contagion as well as infection. Cleanliness and separation are therefore very important measures. It will be obvious to the plainest sense that thorough ventilation is essential.

*Deaths.*—The deaths in the hospital were from the following causes:

| | | | |
|---|---|---|---|
| Apoplexy | 1 | Caries of spine | 1 |
| Bright's Disease | 1 | Rheumatism, acute | 1 |
| Bronchitis | 1 | Diphtheria | 3 |
| Pneumonia | 4 | Erysipelas, phlegmonous | 2 |
| Pleuritis | 1 | Delirium tremens | 1 |
| Phthisis | 3 | *Died after Surgical Operations.* | |
| Diarrhœa, acute | 2 | Amputation of thigh | 1 |
| " chronic | 13 | " " leg | 1 |
| Dysentery | 3 | Trephining skull | 1 |
| Fever, typhoid | 7 | Removal of tumor of neck | 1 |
| Hospital grangrene | 1 | " " vesical calculus | 1 |

*Ratio in some of the important cases.*

| | | | |
|---|---|---|---|
| Pneumonia, | 16 cases, | 4 deaths, | ratio 1 in 4 |
| Pleuritis, | 4 " | 1 " | " 1 " 4 |
| Phthisis, | 13 " | 3 " | " 1 " 4 nearly. |
| Fever, typhoid, | 18 " | 7 " | " 1 " 2½ " |
| Rheumatism, acute, | 10 " | 1 " | " 1 " 10 |
| Delirium tremens, | 17 " | 1 " | " 1 " 17 |
| Diarrhœa, recent, | 20 " | 2 " | " 1 " 10 |
| " chronic, | 25 " | 13 " | " 1 " 2 nearly. |
| Dysentery, | 6 " | 3 " | " 1 " 2 |
| Amputation of thigh | 4 " | 1 " | " 1 " 4 |

It will be observed that the ratio of mortality after amputation of the thigh is less than the average.

Of the three cases of recovery two were after long existing chronic disease, viz: one of caries of the bones about the knee joint, and the other of extensive necrosis of the tibia, involving the articulating head, and accompanied by chronic disease of the cavity of the knee joint. The third was after a severe railroad accident. In the two, the general condition of health was favorable; in the third, the shock of injury was great, and the injury severe. Great suppuration took place. It was a case of secondary amputation, not thought usually, to have much chance of recovery. The recovery was, therefore, quite surprising, from the double shock of the injury and the operation, and the exhausting effects of pain and suppuration. A month's trial to save the limb was made. The fatal case occurred after an amputation for long standing disease of the knee joint, with much swelling and great suppuration. The patient was much worn down by continued and constant pain, and purulent discharge, and survived the operation but a short time.

